# Supplementary material for: Structural basis for inhibition of erythrocyte invasion by antibodies to Plasmodium falciparum protein CyRPA
Source: eLife. 2017 Feb 14;6:e21347. doi: 10.7554/eLife.21347 (PMC5349848; doi:10.7554/eLife.21347)
Supplement: Figure 4—source data 1. — Hydrogen bonds were computed using the program CONTACT within the CCP4 suite (Collaborative Computational Project N, 1994). DOI: http://dx.doi.org/10.7554/eLife.21347.009 [file elife-21347-fig4-data1.docx]

**Figure 4 – source data 1.** Potential hydrogen bonds between CyRPA and Fab 8A7. Hydrogen bonds were computed using the program CONTACT within the CCP4 suite [44]

| **CyRPA** | **Fab 8A7**^1^ |
| --- | --- |
| Glu 42 O | Lys 57H Nζ |
| Lys 66 O | Arg 100H Nη1 |
| Lys 66 Nζ | Asp 92L O |
| Glu 67 Oε1 | Thr 50L Oγ1 |
| Glu 67 Oε2 | Tyr 91L OH |
| Thr 68 O | Arg 100H Nη1 |
| Asp 69 O | Trp 33H N |
| Asp 69 Oδ2 | Val 99H N |
| Asp 69 Oδ2 | Arg 100H N |
| Asp 69 Oδ2 | Arg 100H Nη2 |
| Asp 69 Oδ1 | His 35H Nε2 |
| Asp 69 Oδ1 | Arg 100H Nε |
| Thr 71 Oγ1 | Thr 30H O |
| Thr 71 N | Ser 31H O |
| Glu 91 Oε1 | Ser 31H Oγ |
| Lys 99 Nζ | Ser 28H Oγ |
| Asn 116 Oδ1 | Thr 53L Oγ1 |
| Asn 117 O | Tyr 49L OH |
| Glu 119 Oε2 | Arg 101H Nε |

^1^The residue number suffices H and L denote the respective heavy and light chains of the Fab 8A7.
